# Effusanin B Inhibits Lung Cancer by Prompting Apoptosis and Inhibiting Angiogenesis

**DOI:** 10.3390/molecules28237682

**Published:** 2023-11-21

**Authors:** Jiantong Hou, Ying Li, Honghong Xing, Ruyu Cao, Xiaomeng Jin, Jing Xu, Yuanqiang Guo

**Affiliations:** 1 State Key Laboratory of Medicinal Chemical Biology, Tianjin Key Laboratory of Molecular Drug Research, and College of Pharmacy, Nankai University, Tianjin 300350, China; houjiantong123@163.com (J.H.); liying@baridd.ac.cn (Y.L.); 2120171289@mail.nankai.edu.cn (H.X.); 15369879036@163.com (R.C.); jin20170102@163.com (X.J.); 2 Key Laboratory of Tropical Medicinal Resource Chemistry of Ministry of Education, Hainan Normal University, Haikou 571158, China

**Keywords:** diterpenoid, anti-tumor, STAT3, FAK, effusanin B, angiogenesis, zebrafish

## Abstract

Cancer is one of the deadliest human diseases, causing high rates of illness and death. Lung cancer has the highest mortality rate among all malignancies worldwide. Effusanin B, a diterpenoid derived from *Isodon serra*, showed therapeutic potential in treating non-small-cell lung cancer (NSCLC). Further research on the mechanism indicated that effusanin B inhibited the proliferation and migration of A549 cells both in vivo and in vitro. The in vitro activity assay demonstrated that effusanin B exhibited significant anticancer activity. Effusanin B induced apoptosis, promoted cell cycle arrest, increased the production of reactive oxygen species (ROS), and altered the mitochondrial membrane potential (MMP). Based on mechanistic studies, effusanin B was found to inhibit the proliferation and migration of A549 cells by affecting the signal transducer and activator of transcription 3 (STAT3) and focal adhesion kinase (FAK) pathways. Moreover, effusanin B inhibited tumor growth and spread in a zebrafish xenograft model and demonstrated anti-angiogenic effects in a transgenic zebrafish model.

## 1. Introduction

Cancer is gaining prominence as the leading cause of death. Among the most frequently diagnosed cancers, lung cancer has a high mortality rate in China [1]. So, there is an urgent need for a breakthrough to be made in the medical treatment of lung cancer. Lung cancer can be classified into small-cell lung cancer (SCLC) and non-small-cell lung cancer (NSCLC), with the majority being NSCLC (approximately 85%). Currently, surgical resection, chemotherapy, radiotherapy, targeted therapy, and immunotherapy are the primary treatment options for lung cancer [2]. However, these treatment methods do not fully meet clinical requirements. Chemotherapy drugs are particularly limited in their long-term use due to side-effects and drug resistance. Phytochemicals have demonstrated promising efficacy and fewer side-effects in various diseases, prompting the ongoing search for antitumor drug candidates from plants and other natural sources.

*Isodon serra*, also known as Xihuangcao, is a traditional medicinal plant. It is primarily distributed in Jiangxi, Guangdong, Fujian, and other southern regions of China [3]. This plant is known for its ability to clear heat and detoxify, promote dampness, reduce yellowing, dispel blood stasis, and reduce swelling [4]. According to previous studies, various types of constituents including diterpenoids, phenolics, flavonoids, and glycosides have been found in the medicinal plant *I. serra* [3,5]. Diterpenoids have been reported as the main component of the leaves of *I. serra* [6], exhibiting various biological effects such as cytotoxic activity [7].

During our ongoing investigation of natural substances with antitumor properties in plants, the class of diterpenoids has attracted significant attention. Many diterpenoids isolated from natural sources have been found to exhibit significant antitumor activity. As a well-known example, paclitaxel is a natural diterpenoid that has been utilized as a clinical drug to treat various cancers, including advanced non-small-cell lung cancer, ovarian cancer, and metastatic breast cancer [8,9]. Plant-derived triptolide has been reported to disrupt key molecular signaling pathways in NSCLC, exhibiting cytotoxic activity [10]. Trigothysoid N, a naturally occurring daphnane diterpenoid, has been documented to exhibit anti-tumor properties through the inhibition of A549 cell proliferation [11,12,13,14].

As a diterpenoid, effusanin B was purified from the medicinal plant *I. serra* and was initially documented in 1980 [15]. In previous studies, effusanin B has been reported to exhibit DNA-damaging and cytotoxic activity [16,17]. However, there have been no comprehensive studies on its in vivo antitumor effects and mechanism. In our search for bioactive natural compounds from medicinal plants to combat cancer, effusanin B was isolated and identified from *I. serra*. In our cellular bioassay, effusanin B appeared to have stronger cytotoxicity against A549 cells compared to the positive control etoposide, which indicated that effusanin B may be potentially useful for treating NSCLC. So, the in vivo antitumor effects and mechanism of this compound were further explored and reported.

## 2. Results

**Figure 1:**
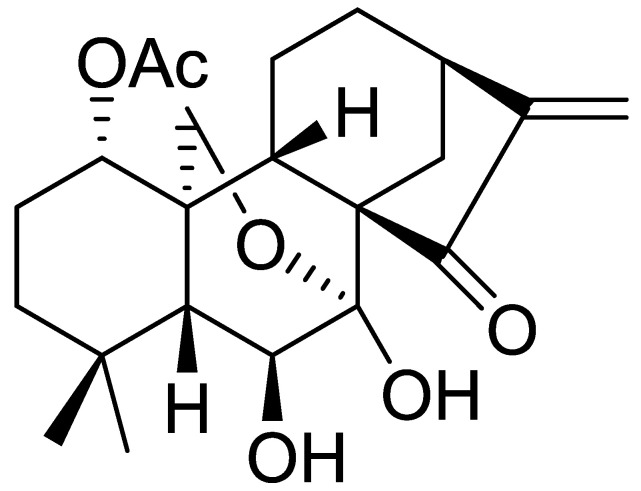
Chemical structure of effusanin B.

Effusanin B was purified from the medicinal plant *I. serra*. The NMR spectrum is provided in the Supplementary Material (Figure S1). By comparing the NMR data with those reported [18], the compound was determined to be effusanin B and its structure was as follows (Figure 1).

### 2.1. Effusanin B Inhibited Tumor Cell Proliferation In Vitro 

In the subsequent biological screening using various tumor cell lines, effusanin B was found to exhibit significant anti-proliferative effects on A549 cells. The 50% growth inhibitory concentration (IC_50_) of effusanin B and etoposide in A549 cells was calculated to be 10.7 µM and 16.5 µM. Effusanin B exhibited a significantly greater cytotoxicity compared to the positive control in a dose- and time-dependent manner (Figure S2). The results suggested that effusanin B had stronger inhibitory effects than the positive control, etoposide.

### 2.2. Apoptotic Effects of A549 Cells Induced by Effusanin B

**Figure 2:**
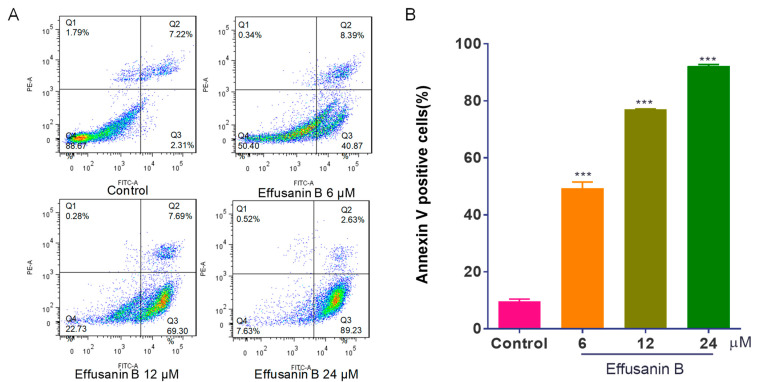
Effusanin B induced apoptosis in A549 cells. A549 cells were exposed to effusanin B (6, 12, and 24 μM) for 48 h. The cells labeled with propidium iodide (PI) and Annexin V were assessed using flow cytometry. (**A**) Flow cytometric analysis of A549 cells treated with various concentrations of effusanin B. (**B**) Histogram showing the proportions of apoptotic cells at 48 h with the treatment of effusanin B. The results are expressed as means ± SD. *** *p* < 0.001 versus the control group.

To confirm the cytotoxic effects and investigate the possible mechanism, the apoptosis-inducing effect of effusanin B was examined. Annexin V-FITC/PI double staining was used. The results indicated that the number of both early and late apoptotic cells increased in a dose-dependent manner. As shown in Figure 2, the percentage of apoptotic cells increased from 9.53% (control) to 49.26% (6 μM), 76.99% (12 μM), and 92.16% (24 μM). The data above indicated that effusanin B induced apoptosis in a concentration-dependent manner. The results demonstrated that effusanin B had cytotoxic effects and induced apoptosis in A549 cells.

### 2.3. Effects of Effusanin B on Cell Cycle

**Figure 3:**
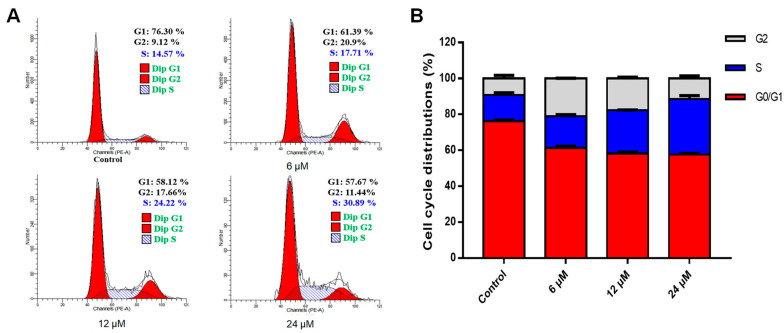
Effusanin B arrested the A549 cells in the S phase. A549 cells were treated with effusanin B (6, 12, and 24 μM) for 48 h. Subsequently, propidium iodide (PI) was used to label the cells, and the cell cycle distribution was analyzed using flow cytometry. (**A**) Flow cytometric analysis of A549 cells after being treated with effusanin B. (**B**) Histogram showing the distribution of cell cycle phases.

Apoptosis is closely related to cell cycle progression and is caused by the termination and disruption of the cell cycle. To investigate whether the apoptosis induced by effusanin B was related to cell cycle arrest, the cell cycle distribution was assessed after treating A549 cells with effusanin B (6, 12, and 24 μM). According to the quantification results shown in Figure 3, the percentage of the S phase increased from 14.57% in the control group to 17.71% (6 μM), 24.22% (12 μM), and 30.89% (24 μM). Taken together, these results suggested that effusanin B could induce S-phase cell cycle arrest in A549 cells.

### 2.4. Effusanin B Induced the MMP

**Figure 4:**
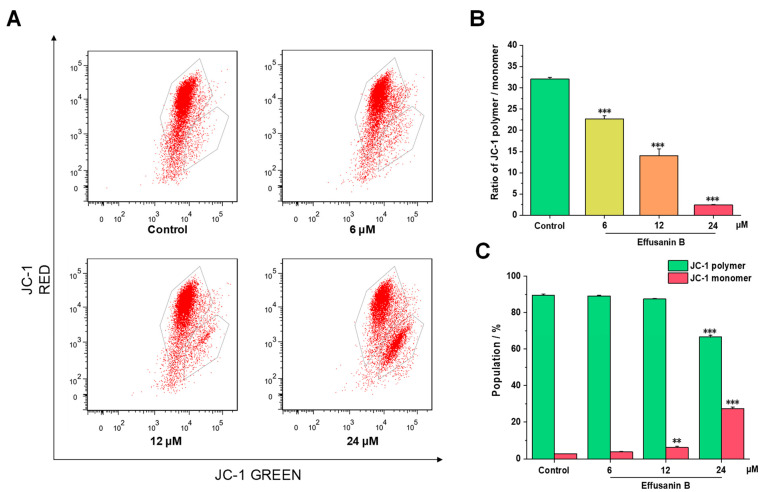
Effusanin B affected MMP levels in A549 cells. A549 cells were treated with effusanin B (6, 12, and 24 μM) for 48 h. Then, the cells were stained with JC-1, and the mitochondrial membrane potential was measured using flow cytometry. (**A**) Flow cytometric analysis of A549 cells after being treated with effusanin B. (**B**) Histogram of the ratio of JC-1 polymer/monomer. (**C**) Histogram of the population of JC-1 polymer and monomer. The results are expressed as means ± SD. ** *p* < 0.01 and *** *p* < 0.001 versus control group.

Generally, mitochondrial membrane potential levels in tumor cells are much higher than those in normal cells [19]. The change in mitochondrial membrane potential plays an important role in the apoptosis of tumor cells, and this imbalance usually indicates cell apoptosis. To investigate whether MMP levels were changed, fluorescent mitochondrial probe JC-1 was used to measure MMP using flow cytometry. From the results shown in Figure 4A, a considerable amount of the red fluorescence was converted to green. As presented in Figure 4B,C, JC-1 polymer and the ratio of JC-1 polymer/monomer significantly decreased as the dose of effusanin B increased. Collectively, these results suggested that effusanin B could induce depolarization of the mitochondrial membrane potential. The depolarization generally contributes to cell apoptosis. 

### 2.5. Effusanin B Induced ROS Generation in A549 Cells

**Figure 5:**
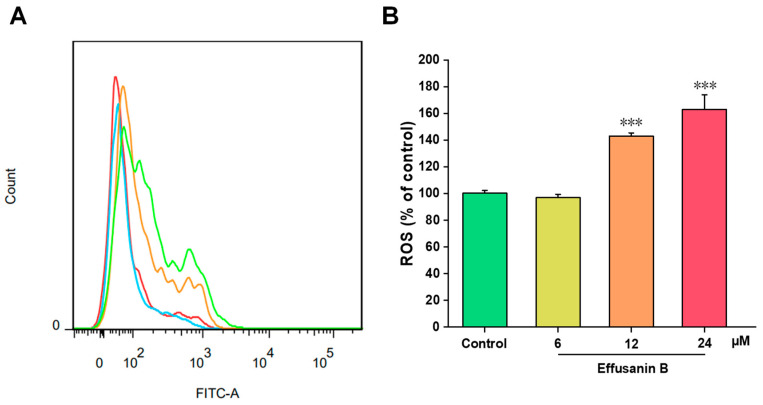
Effusanin B increased ROS levels of A549 cells. A549 cells were treated with effusanin B (6, 12, and 24 μM) for 48 h. PI was used to label the cells, and flow cytometry was used to assess the results. (**A**) Flow cytometric analysis of A549 cells after being treated with different concentrations of effusanin B. (**B**) Histogram of relative ROS level compared with the control group. The results are expressed as means ± SD. *** p < 0.001 versus the control group.

Exogenous stimuli, such as drugs and chemicals, typically result in an elevation in intracellular ROS in mitochondria, which in turn triggers cell apoptosis. To investigate whether effusanin B induced the generation of ROS to initiate cell apoptosis, the levels of ROS were measured. After treatment with effusanin B, the production of ROS in A549 cells was measured using the fluorescent probe DCFH-DA and flow cytometry. As shown in Figure 5, the ROS levels of A549 cells were 1.4-fold (12 μM) and 1.6-fold (24 μM) higher compared to the control group. The results indicated that effusanin B could induce ROS generation to induce apoptosis in A549 cells. 

### 2.6. Effusanin B Affected the Expression of Apoptosis-Related Proteins

**Figure 6:**
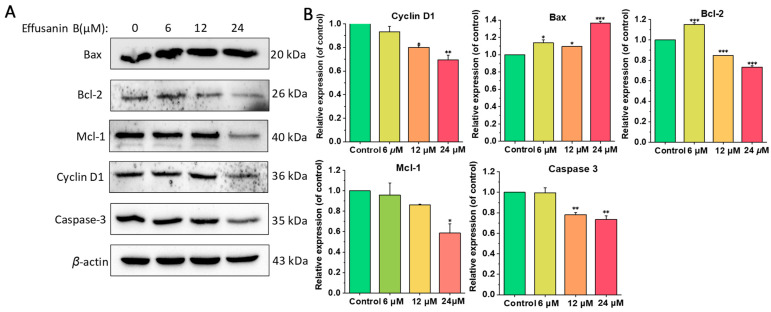
Effusanin B regulated proteins related to apoptosis. (**A**) Western blotting analysis was performed to evaluate the impact of effusanin B on the expression of Bax, Bcl-2, Mcl-1, Cyclin D1, and caspase-3. (**B**) A histogram depicting the relative protein expression levels compared to the control group. The results are expressed as means ± SD. * *p* < 0.05, ** *p* < 0.01, and *** *p* < 0.001 versus control group.

The present study indicated that effusanin B induced apoptosis in a concentration-dependent manner. To further investigate the underlying molecular mechanism, the expression of proteins associated with the mitochondria-dependent apoptotic pathway was examined using Western blotting experiments. As shown in Figure 6, the expression of the pro-apoptotic protein Bax was upregulated with the treatment of effusanin B, and the expression of the anti-apoptotic Bcl-2, Mcl-1, and Caspase-3 protein was significantly downregulated. The changes in protein expression including the above MMP and ROS results showed that effusanin B could alter the mitochondrial membrane potential and stimulate apoptosis in A549 cells.

### 2.7. Effusanin B Regulated STAT3 Signaling Pathway

**Figure 7:**
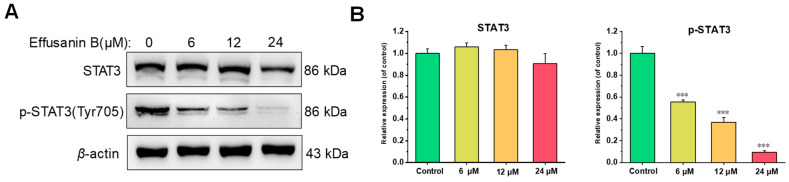
Effusanin B regulated the STAT3 signaling pathway. (**A**) Western blotting analysis was conducted to evaluate the effects of effusanin B on the expression of STAT3 and p-STAT3. (**B**) Histogram showing the relative expression level of the protein compared with the control group. The results are expressed as means ± SD. *** *p* < 0.001 versus the control group.

Signal transducer and activator of transcription 3 (STAT3) has been reported to be associated with tumor progression as a member of the STAT protein family. The STAT3 signaling pathway, including the phosphorylation levels of STAT3 and the downstream protein expression, is directly related to tumor proliferation. To determine the influence of effusanin B on the STAT3 signaling pathway, the expression levels of proteins related to proliferation were measured. Western blotting analysis revealed that effusanin B did not affect the overall expression of STAT3 (Figure 7). However, effusanin B could dose-dependently decrease the levels of STAT3 phosphorylation at Tyr 705 (Figure 7). Furthermore, the current results demonstrated the changes in protein expression levels downstream of STAT3. The levels of Cyclin D1, Mcl-1, and Bcl-2 exhibited a dose-dependent decrease, while the pro-apoptotic protein Bax showed a dose-dependent increase (Figure 6). These findings suggested that effusanin B exerted an anti-proliferative activity in A549 cells by regulating the STAT3 pathway.

### 2.8. Effusanin B Inhibited A549 Cell Metastasis by Regulating the FAK Signaling Pathway

**Figure 8:**
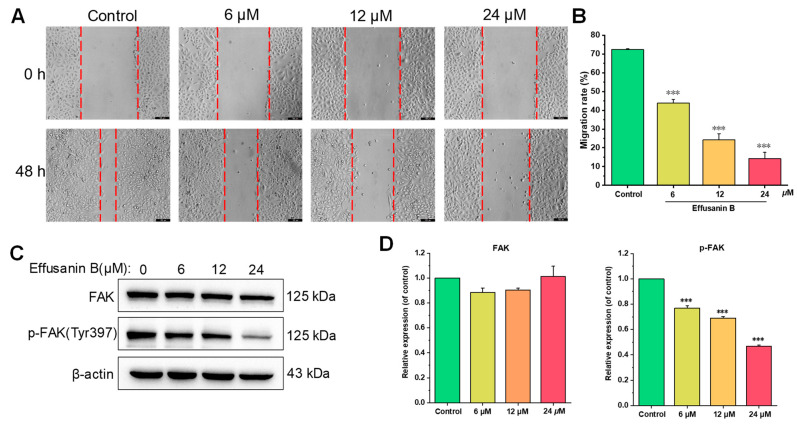
Effusanin B inhibited the tumor migration and regulated the FAK signaling pathway. (**A**) A549 cells were photographed at 0 h and 48 h. (**B**) Data on migration rates (%) are shown in the histogram. (**C**) Western blotting analysis was used to evaluate the effects of effusanin B on the expression of FAK and p-FAK. (**D**) Histogram of the relative protein expression level compared with the control group. The results are expressed as means ± SD. *** *p* < 0.001 versus the control group.

Besides uncontrolled proliferation, migration is also an important characteristic of tumors. Therefore, the inhibitory effects of effusanin B on tumor migration were examined using a wound-healing assay. As shown in Figure 8, after a 48 h treatment with effusanin B, the migration of A549 tumor cells was significantly blocked. The migration rates were 72.43% (control), 43.88% (6 μM), 24.27% (12 μM), and 14.29% (24 μM), respectively.

It is well known that tumor migration is usually regulated by focal adhesion kinase (FAK) and its downstream proteins [20]. The effects of effusanin B on the expression of FAK and the downstream proteins were analyzed. As shown in Figure 8, the total expression of FAK did not change after treatment with effusanin B, while the expression of phosphorylated FAK was significantly inhibited. These results suggested that effusanin B inhibited tumor metastasis by regulating the FAK signaling pathway.

### 2.9. Effusanin B Possessed In Vivo Antitumor Effects

**Figure 9:**
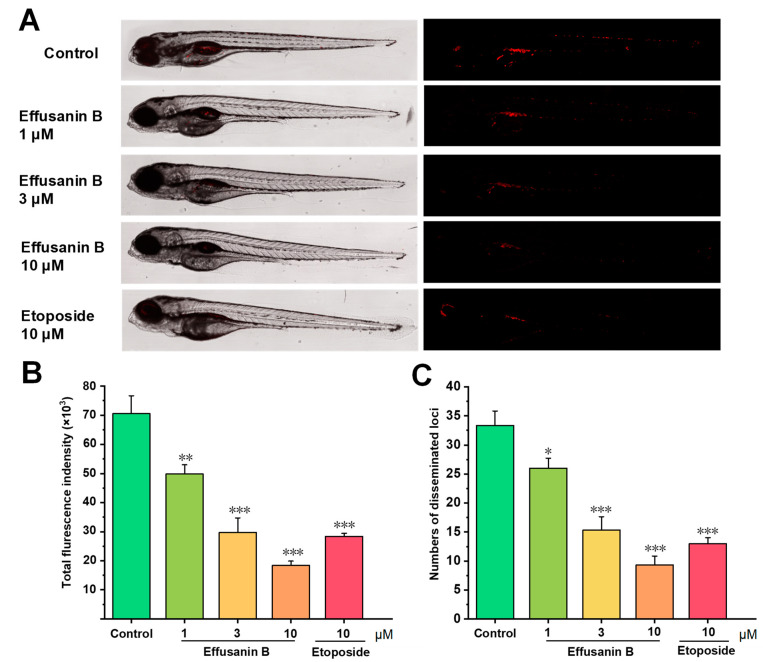
Effusanin B inhibited tumor proliferation and migration in vivo. A549 cells were stained with CM-DiI and then microinjected into 2 dpf zebrafish embryos. After 4 h, the embryos mentioned above were treated with effusanin B (1, 3, and 10 μM) and etoposide (10 μM) for 48 h (15 embryos/group). (**A**) The intensity and distribution of the red fluorescence in disseminated foci in zebrafish were imaged under a confocal microscope. (**B**) The proliferation was quantified using ImageJ software. (**C**) The metastasis of A549 cells was quantified using ImageJ software. All results are expressed as the mean ± SD. * *p* < 0.05, ** *p* < 0.01, and *** *p* < 0.001 versus the control group.

The cellular experiments including tumor cell apoptosis and related mechanisms confirmed that effusanin B exhibited cytotoxic effects and showed potential for cancer treatment. Thus, the in vivo anti-proliferation and anti-metastasis effects of effusanin B were further investigated using a zebrafish xenotransplantation model. After exposing zebrafish to effusanin B (1, 3, and 10 μM), no deformities or deaths were observed, indicating that the compound did not exhibit any obvious toxicity to zebrafish at the tested concentrations (Figure S4). The zebrafish xenograft tumor model was established by microinjecting stained cancer cells into zebrafish, and the fluorescence was analyzed quantitatively. As shown in Figure 9, compared with the blank control, the proliferation and migration of A549 cells were significantly inhibited after treatment with effusanin B and the positive control, etoposide, compared to the blank control. As shown in Figure 9B,C, the relative intensity and focus of red fluorescence decreased dose-dependently. In particular, the inhibition rates of effusanin B at a concentration of 10 µM on the relative intensity and red fluorescence focus were 73.87% and 72.01%, respectively. Taken together, zebrafish xenotransplantation experiments showed that effusanin B had antitumor effects in vivo by inhibiting the proliferation and metastasis of A549 cells.

### 2.10. Effusanin B Blocked Angiogenesis In Vivo

**Figure 10:**
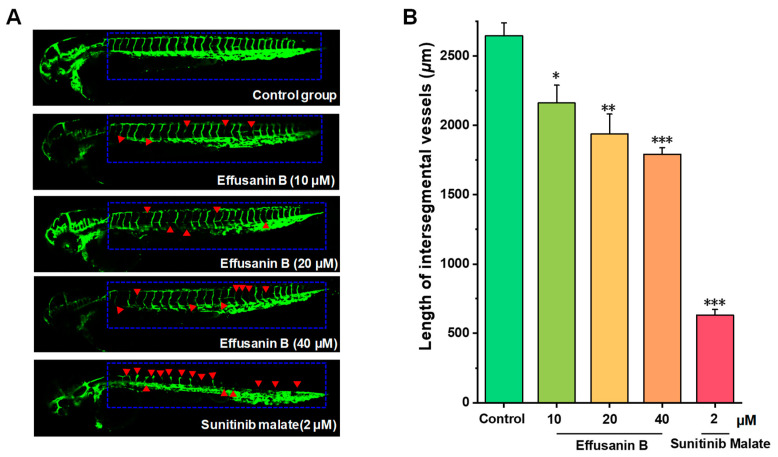
The effects of effusanin B on transgenic zebrafish angiogenesis. The embryos were obtained from transgenic zebrafish *Tg(fli1:EGFP)*. Effusanin B and sunitinib malate (2 μM) were used to treat the embryos mentioned above for 48 h. (**A**) The development of ISVs was observed under a confocal microscope. The red arrows indicated the condition of angiogenesis inhibition. (**B**) The average length of ISVs of zebrafish after treatment with effusanin B (10, 20, and 40 μM) and sunitinib malate (15 embryos/group). All results are expressed as the mean ± SD. * *p* < 0.05, ** *p* < 0.01, and *** *p* < 0.001 versus control group.

Angiogenesis is also a crucial factor in the onset, progression, and spread of tumors. It is well known that newly formed blood vessels provide and transport various nutrients for the proliferation of tumor cells. To investigate the effects of effusanin B on angiogenesis, transgenic zebrafish *Tg(fli1:EGFP)* were used to detect the effects on newly formed blood vessels. As shown in Figure 10, intersegmental vessels (ISVs) of transgenic zebrafish were disrupted after treatment with effusanin B. As shown in Figure 10, the length of the ISVs increased from 2646.6 ± 92.8 μm (control) to 2161.9 ± 128.0 μm (10 μM), 1937.8 ± 144.1 μm (20 μM), and 1791.5 ± 46.9 μm (40 μM). The angiogenesis experiments using transgenic zebrafish showed that effusanin B could inhibit angiogenesis and contribute to tumor suppression.

## 3. Discussion

Cancer is one of the deadliest diseases in humans, causing high rates of illness and death. Lung cancer is the leading cause of death worldwide. Non-small-cell lung cancer (NSCLC) accounts for 80–85% of all cases. Traditional cancer treatments have limited clinical application due to various flaws. Therefore, there is an urgent need to find low-toxicity and efficient antitumor lead compounds for the development of new drugs. The promising efficacy and fewer side-effects of phytochemicals have prompted the search for antitumor drug candidates from natural sources. 

Effusanin B, a natural diterpenoid obtained from the medicinal plant *I. serra*, exhibited greater cytotoxicity than the positive control etoposide against A549 cells. The promising activity of effusanin B on A549 cells has sparked our interest in understanding its therapeutic mechanism and potential application for NSCLC.

Proliferation is one of the main factors contributing to the development and progression of NSCLC. Cancer can be recognized as malignantly proliferating tumor cells. The occurrence and development of tumors can be influenced by regulating the cell cycle and cell cycle regulators [21]. This study showed that effusanin B inhibited the proliferation of A549 cells by arresting the cell cycle at the S phase. Apoptosis is a well-known form of programmed cell death (PCD). The search for new agents that can induce apoptosis is an important direction of tumor treatment. Many studies have focused on compounds that induce apoptosis [22,23,24]. In this study, effusanin B significantly induced apoptosis in a concentration-dependent manner.

There are two core pathways inducing apoptosis including the extrinsic-death receptor pathway and the intrinsic-mitochondrial pathway [25]. Mitochondria is important for cell function and the decision-making steps in cell death [26]. It is also an antitumor strategy by affecting mitochondrial function to induce the intrinsic-mitochondrial apoptosis pathway [27,28]. The Bcl-2 family plays a crucial role in the regulation of apoptotic cell death, which consists of anti-apoptotic members, such as Bcl-2/Bcl-x, and pro-apoptotic members like Bax. Bcl-2/Bax within mitochondria can exert an impact on the release of factors associated with apoptosis, thereby inducing mitochondrial dysfunction, including the loss of membrane potential (Δψ) and the occurrence of the membrane permeability transition [29,30]. Many investigations have reported that Bcl-2 family proteins play an important role in the process of mitochondrial outer membrane permeabilization (MOMP) [31]. MOMP causes the release of Cytochrome C, which further promotes caspase activation and apoptosis [32,33,34]. Mcl-1, an anti-apoptotic protein, is a member of the Bcl-2 family and is involved in maintaining mitochondrial integrity [35]. Our results showed that the expression levels of Bcl-2, Mcl-1, and Caspase 3 were gradually downregulated in a dose-dependent way, while the expression levels of Bax and Cleaved caspase 3 were gradually upregulated after treatment with effusanin B. This study also showed that effusanin B induced the loss of MMP and ROS generation in a dose-dependent manner. Moreover, the activation of the Bcl-2 family can also induce ROS generation [36,37]. The increase in ROS can promote apoptosis by mediating mitochondrial permeability [11,12,38]. The present study indicated that effusanin B induced apoptosis in tumor cells by targeting the mitochondrial pathway and promoting an increase in ROS.

STAT3 is a cytoplasmic transcription factor involved in cell proliferation, metastasis, angiogenesis, inflammation, and immune responses [39]. STAT3 is activated through tyrosine phosphorylation. Then, STAT3 forms dimers to enter the nucleus and regulates the expression of genes related to cell proliferation, invasion, and angiogenesis [40]. The present study showed that the expression levels of p-STAT3, Mcl-1, and Cyclin D1 were gradually downregulated in a dose-dependent manner after treatment with effusanin B. Mcl-1 [41] and Cyclin D1, the downstream target proteins of STAT3 signaling, are associated with cell proliferation [42]. These results indicated that effusanin B inhibited A549 cell proliferation through the STAT3 pathway.

Cell migration is a significant characteristic of NSCLC, in addition to proliferation. In the present investigation, the wound healing assay demonstrated a significant suppression of cell migration when treated with effusanin B. FAK exerts a crucial role in cancer cell proliferation and migration. In the case of unchanged total FAK protein levels, the decrease in FAK activity could inhibit tumor progression and metastasis [19]. The downregulation of FAK signaling is also a therapeutic strategy that suppresses the migration of A549 cells [13,43]. These results from the current study suggested that effusanin B inhibited tumor metastasis by regulating critical proteins in the FAK signaling pathway.

In addition to verifying the antitumor activity against A549 cells in vitro, we also investigated the in vivo antitumor activity of effusanin B using the zebrafish model. The results showed that effusanin B inhibited the proliferation and metastasis of A549 cells in vivo. It is well known that tumor cells require a significant amount of nutrients due to their rapid growth. The nutrients and oxygen necessary for tumor growth can be provided by blood vessels. It is an effective method to inhibit angiogenesis for tumor treatment [44]. Vascular endothelial growth factor (VEGF) plays a critical role in tumor angiogenesis. VEGF can bind to vascular endothelial growth factor receptor (VEGFR), thereby activating a series of downstream pathways that are related to tumor angiogenesis [45]. The present results showed that effusanin B could significantly block angiogenesis.

## 4. Materials and Methods

### 4.1. Materials and Cell Culture

Dulbecco’s Modified Eagle’s Medium (DMEM) and fetal bovine serum (FBS, BI, Israel) were supplied by Lab Biotech Co. Ltd. (Shandong, China). MTT and DMSO were purchased from BioFroxx (Guangzhou, China) and Solarbio Co. Ltd. (Beijing, China), respectively. The cell tracker CM-DiI was provided by Yeasen Biotechnology Co. Ltd. (Shanghai, China). The Annexin V-FITC Apoptosis Detection Kit, Cell Cycle and Apoptosis Kit, Mitochondrial membrane potential assay kit with JC-1, Reactive Oxygen Species Assay Kit, and BCA protein assay kit were obtained from Beyotime Biotechnology Co. Ltd. (Shanghai, China). Mouse monoclonal antibodies against STAT3 and rabbit monoclonal antibodies against Cleaved caspase 3, Caspase 3, Cyclin D1, Mcl-1, Bax, Bcl-2, phospho-STAT3 (Tyr 705), FAK, phospho-FAK, and β-actin were offered by Cell Signaling Technology (Danvers, MA, USA).

The human lung adenocarcinoma cell line (A549) was acquired from Shanghai Institutes for Biological Sciences, Chinese Academy of Sciences (Shanghai, China). The cells were cultured in Dulbecco’s Modified Eagle Medium (DMEM) supplemented with 10% (*v*/*v*) fetal bovine serum and 100 U/mL penicillin/streptomycin, and they were maintained in a water-saturated atmosphere of 5% CO_2_.

### 4.2. Purification of Effusanin B

The details for the extraction, isolation, and purification of effusanin B from the medicinal plant *I. serra* are described in the Supplementary Materials, where the NMR spectrum and data are appended.

### 4.3. Cytotoxicity Assay 

The cytotoxic effects of effusanin B on the A549 cell line were evaluated using the MTT assay [11,12,13,14]. The assay method is supplied in the Supplementary Materials. 

### 4.4. Apoptosis Analysis

The impact of effusanin B on A549 cell apoptosis was assessed using flow cytometry and the Annexin V-FITC Apoptosis Detection Kit (Beyotime, C1062L) [11,12,13,14]. The details are shown in the Supplementary Materials.

### 4.5. Cell Cycle Analysis

The impact of effusanin B on cell cycle distribution was assessed using the Cell Cycle and Apoptosis Kit from Beyotime [11,12,13,14]. The detailed method is supplemented in the Supplementary Materials. 

### 4.6. Mitochondrial Membrane Potential (MMP) Evaluation

Mitochondrial membrane potential (MMP) was measured using an MMP Assay Kit with JC-1 (Beyotime, C2006) following the manufacturer’s instructions [11,12,13,14]. The experimental process is described in the Supplementary Materials.

### 4.7. Measurement of Reactive Oxygen Species (ROS) 

Intracellular production of ROS was detected using the ROS Assay Kit (Beyotime, S0033S) [11,12,13,14]. The measurement method is supplemented in the Supplementary Materials.

### 4.8. Wound-Scratch Assay

The inhibitory effects of effusanin B on the migration of A549 cells were measured using a wound healing assay [11,12,13,14]. The methods and procedures are supplied in the Supplementary Materials. 

### 4.9. Western Blotting Analysis

Western blot analysis was conducted to detect the expression of related proteins using the standard protocols [11,12,13,14]. The details for Western blotting experiments are supplied in the Supplementary Materials. 

### 4.10. Zebrafish Husbandry and Maintenance

Zebrafish were raised in a circulating water system with aeration and ultraviolet sterilization. The zebrafish were fed with commercial fillets twice a day and raised in a 14 h light/10 h dark cycle at a temperature of 28.5 °C. Embryos were obtained using the methods described in previous studies [11,12,13,14]. All the procedures involving animals were approved by the Institutional Animal Care Committee of Nankai University (No. SYXK (JIN) 2019-0001).

### 4.11. In Vivo Antitumor Assay Using Zebrafish Xenografts

An amount of 2 μM of CM-DiI (Yeasen, 40718ES50) was used to label A549 cells. The stained cells were resuspended in serum-free DMEM medium at a density of 1 × 10^7^ cells/mL. An amount of 5 nL prepared cells were injected into the yolk sac of 48 hpf embryos. Then, 4 h later, the randomly divided embryos were treated with varying concentrations (1, 3, and 10 μM) of effusanin B for 48 h. Subsequently, tricaine was used to anesthetize the embryos. The images were captured using a confocal microscope (Leica, Germany), and the red fluorescence density and focus number were quantified using ImageJ software Version 1.54. 

### 4.12. Antiangiogenetic Assay Using a Transgenic Zebrafish Model

The angiogenesis inhibitory activity of effusanin B was assessed using transgenic zebrafish *Tg(fli1:EGFP)* embryos with EGFP-labeled endothelial cells. Adult transgenic zebrafish were acquired from Shanghai FishBio Co., Ltd. (Shanghai, China). Briefly, zebrafish embryos at 6 hpf were randomly distributed into the 12-well plate (15 embryos per group). Various concentrations of effusanin B were administered to the embryos and then incubated at 28.5 °C for 48 h. Subsequently, the zebrafish embryos, anesthetized with 0.02% tricaine, were photographed using a confocal microscope (Leica, Germany). The length of ISV vessels was measured using ImageJ software.

### 4.13. Statistical Analysis

GraphPad Prism 7.0 software was utilized to analyze the experimental data. All data are expressed as the mean ± SD. Significant differences between different groups were compared using one-way ANOVA. Probabilities (P) less than 0.05 indicate a significant difference.

## 5. Conclusions

Recently, scientists have been actively searching for phytochemicals as potential antitumor drug candidates due to their promising efficacy and minimal side-effects. To obtain bioactive substances as lead molecules for anticancer purposes, the extract of *I. serra* was chromatographically purified, and effusanin B was identified. Cellular studies demonstrated that effusanin B inhibited the proliferation and migration of A549 cells by inducing mitochondrial apoptosis and affecting the STAT3/FAK pathways. Mechanistic studies showed that effusanin B inhibited STAT3 phosphorylation and regulated the expression of downstream proteins, such as Bcl-2, Bax, Mcl-1, and Cyclin D1. Effusanin B was found to inhibit cell migration by suppressing FAK phosphorylation. Moreover, effusanin B inhibited the proliferation and metastasis of A549 cells in vivo and demonstrated anti-angiogenic effects in vivo. The experimental results above suggested that effusanin B showed excellent antitumor activity and was expected to be a novel chemotherapeutic agent for NSCLC.

## Data Availability

Data will be made available on request.

## Supplementary Materials

The following supporting information can be downloaded at https://www.mdpi.com/article/10.3390/molecules28237682/s1, Figure S1: ^13^C NMR spectrum of effusanin B; Figure S2: The cytotoxicity in dose and time-dependent manners; Figure S3: The expression of cleavage caspase-3; Figure S4: The survival rate of zebrafish.
