# Supplementary material for: Effusanin B Inhibits Lung Cancer by Prompting Apoptosis and Inhibiting Angiogenesis
Source: Molecules. 2023 Nov 21;28(23):7682. doi: 10.3390/molecules28237682 (PMC10707445; doi:10.3390/molecules28237682)

Supplementary material for

Effusanin B inhibits lung cancer by prompting apoptosis and inhibiting angiogenesis

Contents

1. The extraction and isolation of effusanin B

2. The NMR spectrum of effusanin B

3. Anti-proliferation assay

4. Apoptosis analysis via flow cytometry

5. Cell cycle analysis

6. Mitochondrial membrane potential (MMP) evaluation

7. The measurement of reactive oxygen species (ROS)

8. Wound-scratch assay

9. Western blotting analysis

10. The cytotoxicity in dose and time-dependent manners

11. Effusanin B increased the expression of cleavage caspase-3

12. Developmental toxicity of effusanin B in zebrafish embryos

1. Extraction and isolation of effusanin B

The aerial parts of *I. serra* were purchased from Anguo Herbal Medicine Market in Hebei Province, the People's Republic of China, in April 2018. The plant material was extracted with MeOH under reflux, and the resulting solution was concentrated in vacuo to yield a residue. This residue was dissolved in H_2O and then partitioned successively with petroleum ether (PE) and ethyl acetate (EA). The PE-soluble portion (267.2 g) was subjected to silica gel CC (silica gel, 200–300 mesh, 1200 g; column size, 10 cm × 50 cm) using a gradient elution of PE-acetone (100: 0, 100: 2, 100: 4, 100: 6, 100: 9, 100: 12, 100: 17, 100: 25, and 100: 32) as the eluent. This process yielded nine fractions, A–I, based on TLC analysis. Fraction G (20.0 g) was subjected to the same MPLC protocol using an eluent comprising 62–91% MeOH in H_2O, which resulted in the formation of eight subfractions, G1–G8. Fraction G3 was then purified using preparative HPLC (YMC-pack ODS-AM column, 20 mm × 250 mm) to obtain effusanin B with an eluent of 72% MeOH in H_2O.

2. The NMR spectrum of effusanin B

Fig. S1. ^{13}C NMR spectrum of effusanin B

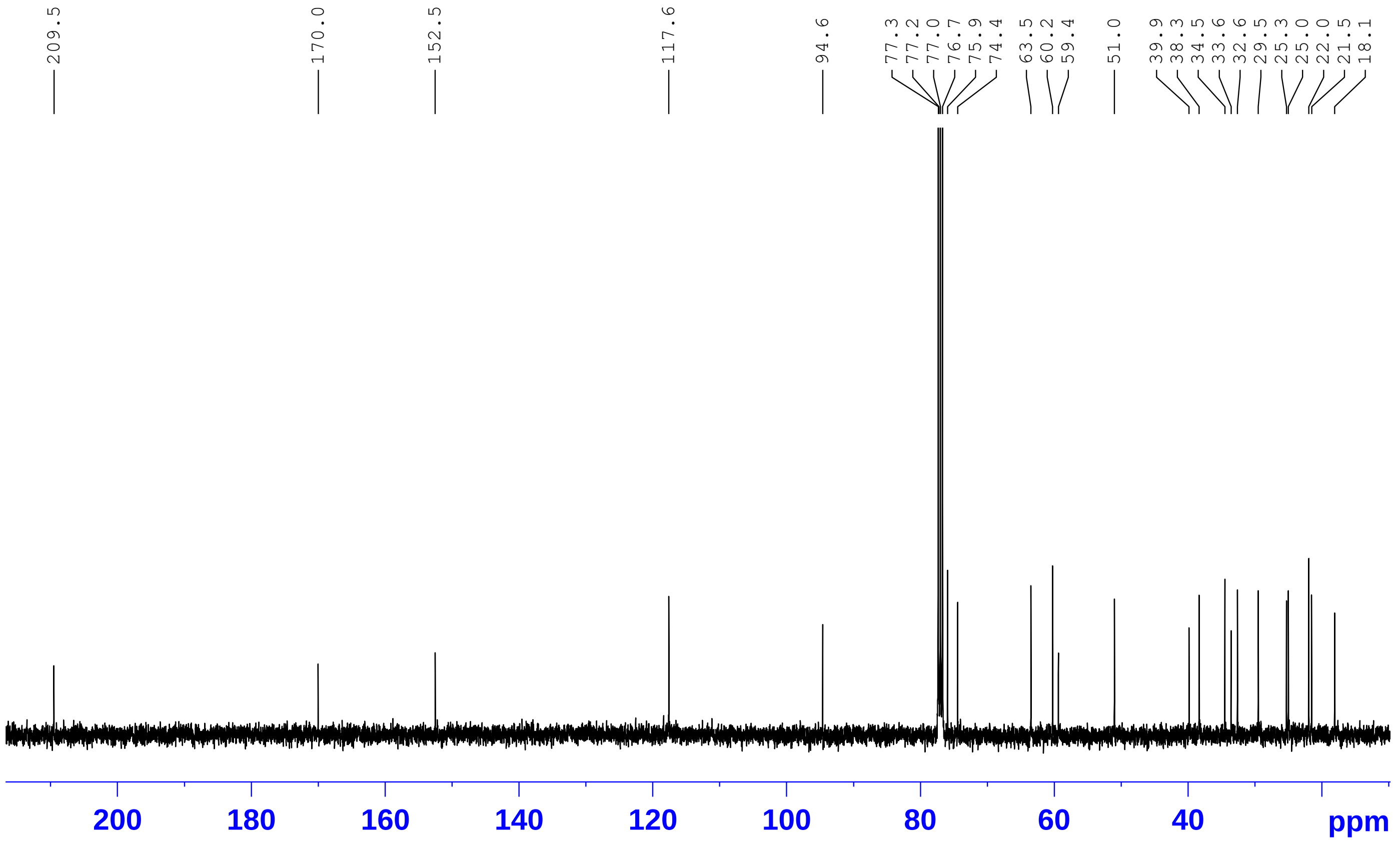

3. Anti-proliferation assay

A549 cells were seeded in 96-well plates with a 100 μL culture medium per well (5000 cells/well). After incubation for 24 h, the cells were treated with various concentrations of effusanin B dissolved in DMSO and were further incubated for 48 h. Then, 20 μL of the MTT solution (5 mg/mL) was added to each well for another 4 h. Subsequently, the medium was sucked away, and 150 μL of DMSO was added into each well to dissolve the formazan that had formed. The absorbance was measured at 492 nm using a microplate reader (Thermo Fisher Scientific Inc. America). The experiments were performed in triplicate, and the IC_{50} value was defined as the concentration of the compounds that inhibited cell proliferation by 50%.

4. Apoptosis analysis via flow cytometry

A549 cells were seeded at a density of 1 × 10^5 cells/well into a 12-well plate and allowed to adhere for 24 h at 37 °C. Then, the cells were incubated with various concentrations (6, 12, and 24 μM) of effusanin B for 48 h before they were harvested and resuspended in the binding buffer. After staining with 5 μL Annexin V-fluorescein isothiocyanate (FITC) and 10 μL phenylindole (PI), the cells were incubated for 20 min. Finally, cell apoptosis was examined using BD LSRFortessa flow cytometry (BD Biosciences). Cell apoptosis data were processed using flow cytometry analysis software (FLOWJO LLC, Ashland, OR, USA).

5. Cell cycle analysis

In detail, A549 cells were passaged in 12-well plates at a density of 1 × 10^5 cells/well. After 24 h, the cells were exposed to various concentrations of effusanin B (6, 12, and 24 μM) for 48 h. Then, the cells were collected and fixed with ice-cold 70% ethanol at 4 °C for 24 h. Next, the cells were stained with a propidium iodide staining buffer containing RNase (Beyotime, C1052) for 30 min at 37 °C in the dark. Subsequently, the phase distribution of the cell cycle was detected using BD LSRFortessa flow cytometry, and the data were analyzed with ModFit LT Software.

6. Mitochondrial membrane potential (MMP) evaluation

Briefly, cells were seeded in 12-well plates at a density of 1 × 10^5 cells/well for 24 h. After treatment with increasing doses (6, 12, and 24 μM) of effusanin B for 48 h, the cells were harvested and then incubated with a JC-1 staining solution for 20 min at 37 °C in the dark. After that, the solution was removed and washed twice with the JC-1 buffer. Finally, the results were analyzed using BD LSRFortessa flow cytometry, and mitochondrial depolarization was evaluated by measuring the decrease in the polymer/monomer (red/green) fluorescence intensity ratio.

7. Measurement of reactive oxygen species (ROS)

In brief, 1 × 10^5 cells were plated in 12-well plates and treated with different concentrations (6, 12, and 24 μM) of effusanin B for 48 h. The cells were collected and stained with 10 μM DCFH-DA at

37℃ for 20 min in the dark. After incubation, the loaded cells were washed three times with serum-free DMEM. The ROS levels were immediately monitored using BD LSRFortessa flow cytometry (BD Biosciences).

8. Wound-scratch assay

A549 cells were seeded into a 6-well plate with a density of 5×10^5 cells/well and were cultured until they attained above 90% confluence as a monolayer. Then, each well was scratched using a sterile pipette tip. Next, different concentrations of effusanin B were added to each well. The closure of the wound gap was photographed at 0 h and 48 h. The scratch area was measured using ImageJ software.

9. Western blot analysis

A549 cells were seeded in a 6-well plate at a density of 2×10^5 cells/well for 24 h and incubated with effusanin B for 48 h. Total proteins were extracted using the RIPA lysis solution (Beyotime, P0013B) and quantified using a BCA protein assay kit (Beyotime, P0012S). The protein samples were separated via 10% SDS-PAGE and then transferred to a polyvinylidene difluoride membrane (Merck Millipore, Darmstadt, Germany). After blocking with 5% skimmed milk for 1 h at room temperature, the membranes were incubated with specific primary antibodies at 4 °C overnight. After washing with TBST, the membranes were subsequently incubated with HRP-conjugated goat anti-mouse/rabbit IgG antibodies for 1 h at room temperature. Finally, the ECL luminescent solution was used to observe the protein blots in the chemiluminescence imager, and the bands were quantitatively analyzed using ImageJ.

10. The cytotoxicity in dose and time-dependent manners

Fig. S2. The cytotoxicity in dose and time-dependent manners

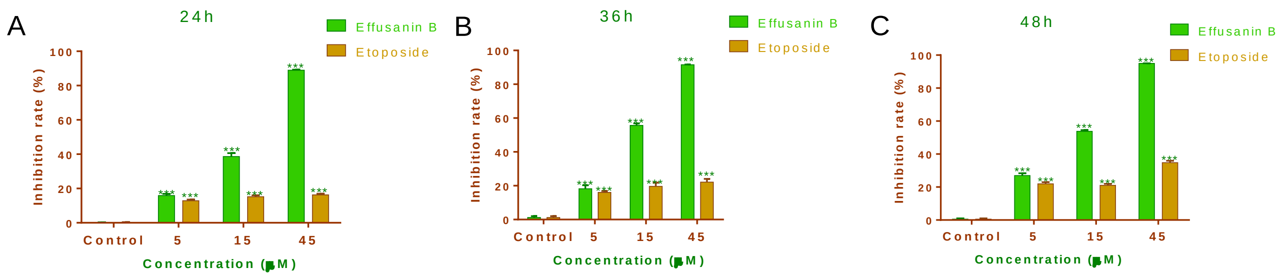

11. Effusanin B increased the expression of cleavage caspase-3.

Fig. S3. The expression of cleavage caspase-3

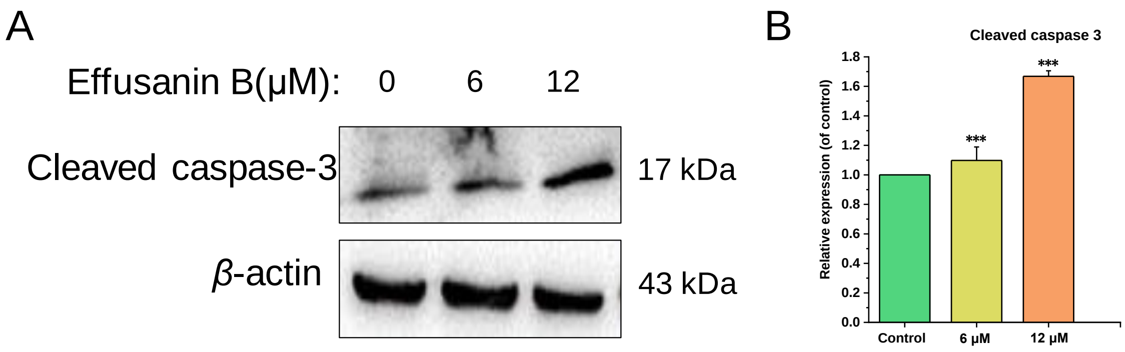

12. Developmental toxicity of effusanin B in Zebrafish Embryos

Fig. S4. The survival rate of zebrafish

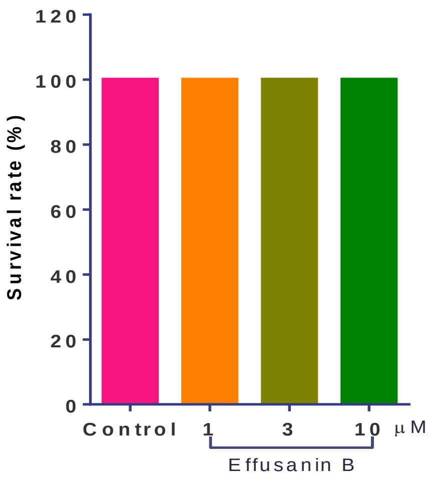

Supplement: Supplementary file 1 [file molecules-28-07682-s001.zip › molecules-2623924-supplementary.pdf]
